# Microbiome variation at the clam-sediment interface may explain changes in local productivity of *Chamelea gallina* in the North Adriatic sea

**DOI:** 10.1186/s12866-023-03146-8

**Published:** 2023-12-19

**Authors:** Giulia Trapella, Nicolò Cinti, Luca Parma, Antonina De Marco, Andrea Nicolò Dell’Acqua, Silvia Turroni, Simone Rampelli, Daniel Scicchitano, Letizia Iuffrida, Alessio Bonaldo, Silvia Franzellitti, Marco Candela, Giorgia Palladino

**Affiliations:** 1 Unit of Microbiome Science and Biotechnology, Department of Pharmacy and Biotechnology (FaBiT), Alma Mater Studiorum – University of Bologna, Bologna, 40126 Italy; 2 Fano Marine Center, The Inter-Institute Center for Research on Marine Bioaffiliationersity, Resources and Biotechnologies, Fano, 61032 Italy; 3 Department of Veterinary Medical Sciences, University of Bologna, Ozzano Emilia (Bologna), 40064 Italy; 4 Animal and Environmental Physiology Laboratory, Department of Biological, Geological and Environmental Sciences (BiGeA), University of Bologna, Ravenna, 48123 Italy

**Keywords:** Bivalves, Host-microbiota interactions, Adriatic Sea, Biodiversity preservation, Metagenomics

## Abstract

**Background:**

The clam *Chamelea gallina* is an ecologically and economically important marine species in the Northwestern Adriatic Sea, which currently suffers from occasional, and still unexplained, widespread mortality events. In order to provide some glimpses in this direction, this study explores the connections between microbiome variations at the clam-sediment interface and the nutritional status of clams collected at four Italian production sites along the Emilia Romagna coast, with different mortality incidence, higher in the Northern sites and lower in the Southern sites.

**Results:**

According to our findings, each production site showed a peculiar microbiome arrangement at the clam-sediment interface, with features that clearly differentiate the Northern and Southern sites, with the latter also being associated with a better nutritional status of the animal. Interestingly, the *C. gallina* digestive gland microbiome from the Southern sites was enriched in some health-promoting microbiome components, capable of supplying the host with essential nutrients and defensive molecules. Furthermore, in experiments conducted under controlled conditions in aquaria, we provided preliminary evidence of the prebiotic action of sediments from the Southern sites, allowing to boost the acquisition of previously identified health-promoting components of the digestive gland microbiome by clams from the Northern sites.

**Conclusions:**

Taken together, our findings may help define innovative microbiome-based management strategies for the preservation of the productivity of *C. gallina* clams in the Adriatic Sea, through the identification and maintenance of a probiotic niche at the animal-sediment interface.

**Supplementary Information:**

The online version contains supplementary material available at 10.1186/s12866-023-03146-8.

## Background

The striped venus clam *Chamelea gallina* is one of the most important fish resources of the Northwestern Adriatic Sea [1], with a reported annual production of 15,000 tonnes in 2018 [2] and a turnover of 100 million euros per year [3]. From an ecological point of view, *C. gallina* is considered an “ecosystem engineer”, capable of influencing nutrient cycling, acting as a habitat-forming species, and affecting food webs directly, as a prey and filter feeder, or indirectly, through sediment movement and nutrient release [4]. Although particularly abundant in the Northwestern Adriatic Sea, *C. gallina* is also found along the Eastern Atlantic coast, from Norway to the Iberian Peninsula, along the coast of Morocco and the Canary Islands, and in the Black Sea. This relatively wide distribution, together with its important ecological role and relevant economical value, makes this clam a very important marine organism [5–7].

*C. gallina* is a typical example of an infaunal microphagous filter feeder [3]. This clam inhabits sandy coasts, occupying a well-defined ecological niche characterized by the presence of specific chemical-physical conditions in water and sandy or muddy-sandy sediments, such as high oxygen content, a redox potential < 300 mV, and a medium to high hydrodynamic regime [2, 8, 9]. The annual reproductive cycle of *C. gallina* is characterized by external planktonic fertilization followed by a larval phase, living in suspension for 15–30 days. After this period, the larvae begin to deposit calcium carbonate in the shell, resulting in sinking and hollowing into the sediment, where the clam settles in a vertical position with siphons protruding from the substrate. The clam then starts its typical benthic life, feeding on organic matter and small planktonic organisms from the water column [3, 10]. The growth of *C. gallina* is relatively fast and, specifically in the Adriatic Sea, clams can reach a diameter of 1.6–1.8 cm after one year, corresponding to their sexual maturity. After about 2 years, they reach the European commercial size of 2.5 cm [3, 11].

Due to its peculiar ecological niche, *C gallina* can only be harvested directly from natural beds. This highlights the strategic importance of implementing measures to protect natural clam populations as the only possible solution for the sustainable management of this valuable marine resource. This topic is particularly relevant in the Adriatic Sea, where *C. gallina* is already suffering a severe decline due to high fishing pressure [12] and several occasional mortality events have been reported [3, 12, 13]. Interestingly, these mortality events showed a local declination to specific sites along the Adriatic coast, that show a higher but irregular incidence of mortality [3]. Although still unexplained, several factors might be involved in the local mortality events observed so far, such as temperature fluctuations, oxygen deficiency, increased water turbidity, and release of xenobiotic compounds [14, 15]. However, the possible involvement of other as yet unexplored factors has been suggested, such as down-regulation of genes typically involved in immune defense or variations of host-microbial interactions in response to environmental changes [3].

Like all marine animals, clams live in close association with a complex microbial community inhabiting their digestive gland (DG) [16]. The DG-associated microbiome plays an integral role in the host biology, providing essential physiological services that are strategic for the clam health [3]. The DG-associated microbiome is involved in nutritional and immunological functions, it can contribute to the enhancement of the defense systems against pathogens and xenobiotics and allows for a better adaptation of the host to stressful conditions [3, 17–19]. Conversely, alterations in the clam DG-associated microbiome have been associated with increased susceptibility to opportunistic infections [3].

**Fig. 1:**
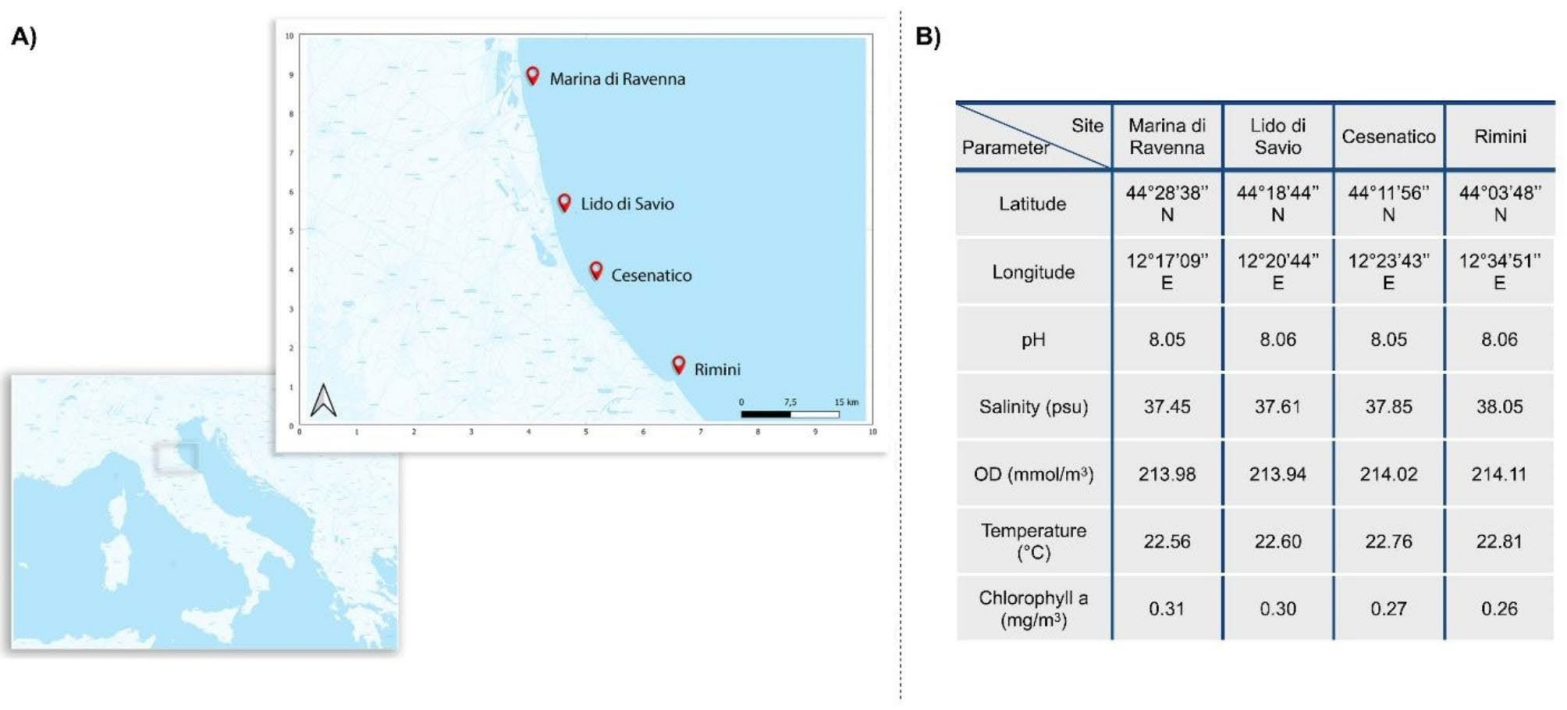
**Sampling sites and environmental parameters. A**) Map showing the sampling sites in Marina di Ravenna (RA), Lido di Savio (RA), Cesenatico (FC) and Rimini (RN), located inside the Flag area along the Emilia Romagna coast. Sampling sites are represented as red dots (map created using the Free and Open Source QGIS at https://www.qgis.org/it/site/). **B**) Environmental parameters on the day of sampling at the 4 sites. Abbreviations: psu = practical salinity unit; OD = dissolved molecular oxygen. Data were retrieved from Copernicus website. (https://data.marine.copernicus.eu/product/MEDSEA_ANALYSISFORECAST_BGC_006_014/description and https://data.marine.copernicus.eu/product/MEDSEA_ANALYSISFORECAST_PHY_006_013/description)

In our work, we collected clam and sediment samples from 4 productive sites along the Northwestern Adriatic coast, Italy. The selected sites were distributed along a gradient from high to low incidence of recorded mortality events and productivity, covering a stretch of 60 km along the coast of the Emilia Romagna region [2, 3, 12, 13] (Fig. 1). We explored possible connections between site-specific features of the DG-associated microbiome of *C. gallina* and local variations in animal nutritional status. Furthermore, as the importance of the interaction between clam DG and sediment microbiome has recently been stressed [3], controlled aquaria experiments were performed, allowing us to elucidate possible mechanisms of interaction between clam-associated microbiomes and sediment microbiomes. By providing some clues on the importance of the interaction between sediment and clam DG-associated microbiomes for animal health and physiological status, our findings pave the way for new microbiome-based approaches and technologies for biomonitoring and conservation of *C. gallina* clams in the Adriatic Sea, with a view to protecting and possibly restoring local populations subject to mortality events.

## Methods

### Sampling area and sample collection

*C. gallina* specimens were collected in four marine sites located 0.4 km off the coast of Marina di Ravenna (Latitude 44°28’38’’N, Longitude 12°17’09’’E), Lido di Savio (44°18’44’’N, 12°20’44’’E), Cesenatico (44°11’56’’N, 12°23’43’’E) and Rimini (44°03’48’’N, 12°34’51’’E), Italy. Sampling was performed by professional local fishermen during the summer season (September 2022) (Fig. 1). For each sampling site, 65 clams were collected for microbiological and physiological analysis, together with the corresponding sediment (50 g) and seawater (2 L). Clam individuals were collected at commercial size (min. 25 mm) by hydraulic dredge, while sediment and seawater were sampled with a Van Veen grab and a Niskin bottle, respectively. After collection, samples were stored at + 4 °C and transported to the laboratory, where they were immediately processed for biometric measurements and DG sectioning. The individual weight of the shells and fresh tissues was assessed using a precision scale (SAUTER Re 2012 Precision Electronic Weighing Scales, METTLER TOLEDO Instruments, Milano, Italy), while the individual length and width of the shell were measured using a caliper (sensitivity ± 0.05 mm, Borletti CDJB20 digital caliper, LTF, Bergamo, Italy). These data were used to calculate the condition index according to Kanduč et al. (2018) [3]. DGs were stored at -80 °C until further processing.

### Proof-of-concept experiment in controlled environment

A proof-of-concept experiment was carried out under controlled conditions at the laboratory of Aquaculture at the Department of Veterinary Medical Sciences of the University of Bologna (Cesenatico, Italy). Four 70-liter aquaria were set up in the laboratory, and filled with recirculating natural Adriatic seawater at a salinity of 23–25 ppt and a temperature of 20 ± 0.5 °C. Each aquarium was independent from the others, with its own electronic heat exchangers and a filtration unit system composed of biological, mechanical and UV filters. About 10 kg of sand from Rimini and Marina di Ravenna was used to fill a 10-cm layer in each aquarium (two aquaria with Ravenna sediments and two with Rimini sediments). From each sampling area, a total of 120 adult clams were collected and transferred to the aquaculture laboratory within 6 h. Sixty animals from Rimini were randomly distributed into an aquarium with Rimini sediments, while the other 60 clams were placed with Ravenna sediments. The same distribution criteria were used for the Ravenna clams. The clams were fed daily with a maintenance ration of live microalgae *Isochrysis galbana*, consisting of 3% of the mean dry-meat weight of the animals in dry weight of algal feed per day, in accordance with the FAO hatchery protocol for bivalve culture [3]. After 3 days of acclimatization, the clams were reared on the different sediments for 21 days.

Clams from the four aquaria were sampled at three different time points (T0, immediately after experimental set-up and acclimatization; T1, after the first 7 days of incubation; T2, after 21 days) for characterization of the DG-associated microbiota, whereas biometric parameters were assessed at T0 and T2. Individual weight of the shells and fresh tissues was assessed using a precision scale (SAUTER Re 2012 Precision Electronic Weighing Scales, METTLER TOLEDO Instruments), while individual length and width of the shell were measured using a caliper (sensitivity ± 0.05 mm, Borletti CDJB20 digital caliper, LTF). The condition index (CI) for clams in controlled environments was calculated according to Kanduč et al. (2018) [3].

### Sample processing and microbial DNA extraction

For field production sites and aquarium-controlled conditions, DGs of *C. gallina* were pooled on base 3 to obtain 12 clam pools per site and 3 to 5 pools for aquarium (4 different aquaria conditions, with Ravenna clams reared on either Ravenna or Rimini sediments and the same for Rimini clams, for 3 different timepoints) for DNA extraction. Microbial DNA was extracted using the DNeasy PowerSoil kit (Qiagen, Hilden, Germany) according to manufacturer’s instructions with minor adjustments. Briefly, the homogenization step was performed using a FastPrep instrument (MP Biomedicals, Irvine, CA, USA), and the elution step was preceded by a 5-min incubation at 4 °C [3, 20]. Seawater samples were processed by vacuum filtration under sterile conditions using 0.22-µm pore size MF-Millipore (Darmstadt, Germany) membrane filters. Seawater microbial DNA was extracted using the Dneasy PowerWater Kit (Qiagen, Hilden, Germany) following the manufacturer’s instructions. Finally, 0.28–0.30 g of sediment from each collection site was used for microbial DNA extraction using the Dneasy PowerSoil Kit (Qiagen) following the manufacturer’s instructions. The extracted DNA was quantified using NanoDrop ND-1000 (NanoDrop Technologies, Wilmington, DE, USA) and stored at -20 °C until further processing.

### 16 S rRNA gene amplification and sequencing

Library preparation was performed according to Illumina 16 S Metagenomic Sequencing Library Preparation protocol (Illumina, San Diego, CA, USA). The V3-V4 hypervariable region of the 16 S rRNA gene was amplified by PCR in 50 µL final volume, containing 25 ng of microbial DNA, 2X KAPA HiFi HotStart ReadyMix (Roche, Basel, Switzerland), and 200 nmol/L forward 314 and reverse 785 primers carrying Illumina overhang adapter sequences [21]. The PCR thermocycle consisted of 3 min at 95 °C, then 30 cycles of 30 s at 95 °C, 30 s at 55 °C and 30 s at 72 °C, and a final elongation step at 72 °C for 5 min [20, 22]. Amplified products were purified using Agencourt AMPure XP magnetic beads (Beckman Coulter, Brea, CA, USA). Indexed libraries were prepared by limited-cycle PCR using Nextera technology (Illumina) and purified again as described above. The libraries were then quantified using a Qubit 3.0 fluorimeter (Invitrogen, Waltham, MA, USA), normalized to 4 nM, and pooled. Finally, the library pool was denatured with 0.2 N NaOH and diluted to 4.5 pM with a 20% PhiX control. Sequencing was performed on an Illumina MiSeq platform using a 2 × 250-bp paired-end protocol, according to the manufacturer’s instructions (Illumina).

### Bioinformatics and biostatistics

Condition index data were analyzed by one-way ANOVA, followed by Tukey’s HSD test (*p*-value ≤ 0.05) for multiple comparisons, after checking the assumptions for ANOVA: Shapiro-Wilk test was used for normality (*p*-value = 0.07) [23], Bartlett’s test for homogeneity of variances (*p*-value = 0.07) [24].

Raw sequences from field-collected samples, totaling 56 samples (12 clam pools per site, 4 sediment and 4 seawater samples), were processed using a combination of the PANDAseq [25] and QIIME 2 pipelines [3]. The “fastq filter” function of the Usearch11 algorithm [26] was applied to retain high-quality sequences. Specifically, based on the probabilities of the phred Q score, sequences with an expected error per base E = 0.03 (i.e., three expected errors per 100 bases) or higher were discarded. High-quality sequences were then clustered into amplicon sequence variants (ASVs) using DADA2 [27]. Taxonomic assignment was performed using a hybrid method combining the VSEARCH algorithm [28] and the q2-feature-classifier plugin [29] trained on the SILVA database (2022, v138.1) [30]. All sequences assigned to eukaryotes or not assigned were discarded. Alpha diversity was calculated by Shannon diversity, Faith’s phylogenetic diversity (PD) and the number of observed ASVs for microbial richness. Beta diversity was calculated using weighted and unweighted UniFrac distances.

All statistical analyses were performed using the R software (R Core Team; http://www.r-project.org), v.4.1.2, with the packages “vegan” (https://cran.r-project.org/web/packages/vegan/index.html), “KEGGREST”(v1.36.3,http://www.bioconductor.org/packages/release/bioc/html/KEGGREST.html), and “gplots” (v3.1.3, https://cran.r-project.org/web/packages/gplots/index.html). UniFrac distances were plotted using the vegan package, and the data separation in the Principal Coordinates Analysis (PCoA) was tested using a permutation test with pseudo-F ratios (function “adonis” in vegan, 999 permutations). Wilcoxon rank-sum test was used to assess significant differences in alpha diversity. *P*-values were corrected for multiple testing using the Benjamini–Hochberg method, with a false discovery rate (FDR) ≤ 0.05 considered statistically significant.

Linear discriminant analysis (LDA) effect size (LEfSe) [31], aimed at identifying discriminant taxa between high- and low-CI sites, was performed on ASV relative abundance tables, retaining only taxa with LDA score threshold of ± 2 (on a log10 scale) and *p*-value ≤ 0.05. The online Galaxy Version interface (https://huttenhower.sph.harvard.edu/galaxy/, last accessed May 2023) was used to run LEfSe. We then used BLAST (last accessed May 2023) [32] to identify bacterial species corresponding to ASV sequences belonging to the discriminant taxa identified by LEfSe.

PICRUSt2 [33] with default parameters was used to predict metagenome functions based on the ASVs identified in our dataset. The output file with the predicted KO (KEGG orthology) copy number per ASV was then used to construct the heatmaps representing the metagenome functions, grouped by pathway, for the discriminant ASVs identified by LEfSe. Reads mapping was performed using Bowtie2 v. 2.3.4.3 [34] for the alignment, with the following parameters “--end-to-end --very-sensitive”. The number of aligned reads for each sample was then retrieved using Samtools v. 1.16.

## Results

### Sample collection, environmental data, and assessment of condition index

**Fig. 2:**
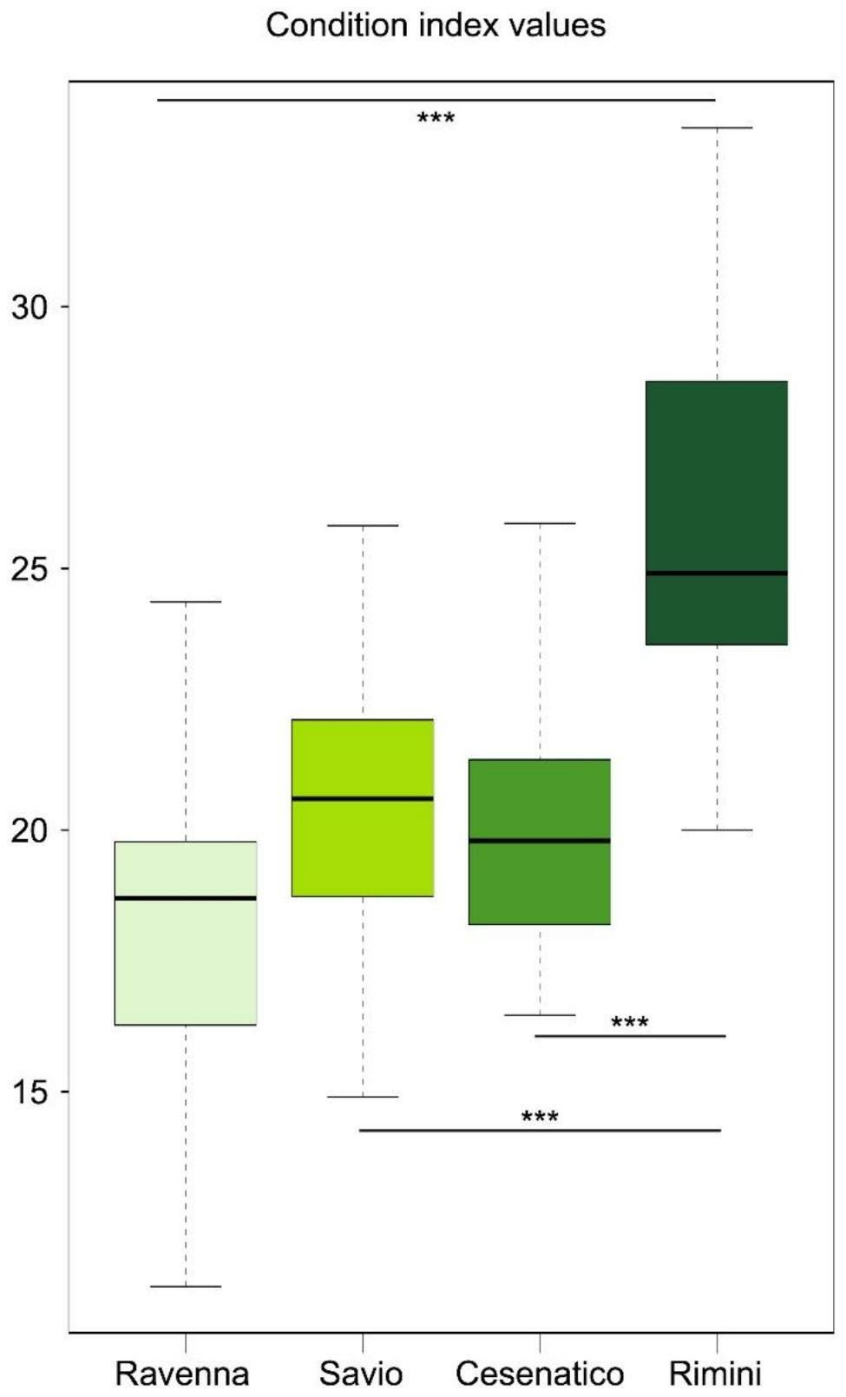
**Condition index values for all sampling locations.** Condition index (CI) was calculated for clams collected at all sampling sites. One-way ANOVA test was used to assess significant differences in CI values among sites (F value = 10.7 and total degrees of freedom = 76)

*C. gallina* samples were collected from 4 different production sites along the Western coast of the North Adriatic Sea (Fig. 1A). Sampling was carried out during the summer season (September 2022) on the same day at all sites, to avoid temporal variations in environmental conditions during the sampling campaign. For each site, 65 clams and a corresponding seawater and sediment sample per site were collected, for a total of 260 clam individuals, 4 seawater samples and 4 sediment samples. Environmental parameters on the day of sampling are reported in Fig. 1B. A total of 20 clams per site were used for the assessment of the Condition Index (CI) as a proxy for the general nutritional and health state of the animal [35], with higher CI values corresponding to a better health status of the clam. The CI values showed a decreasing trend from the Southern to the Northern sites, with Rimini having a CI score significantly higher than the other sites and Marina di Ravenna, Lido di Savio, and Cesenatico having comparable values (one-way ANOVA) (Fig. 2). According to these data, Rimini was considered the only high-CI site, while Marina di Ravenna, Lido di Savio, and Cesenatico were considered low-CI sites.

### Core and variable fractions of the clam digestive gland-associated and sediment microbiomes

**Fig. 3:**
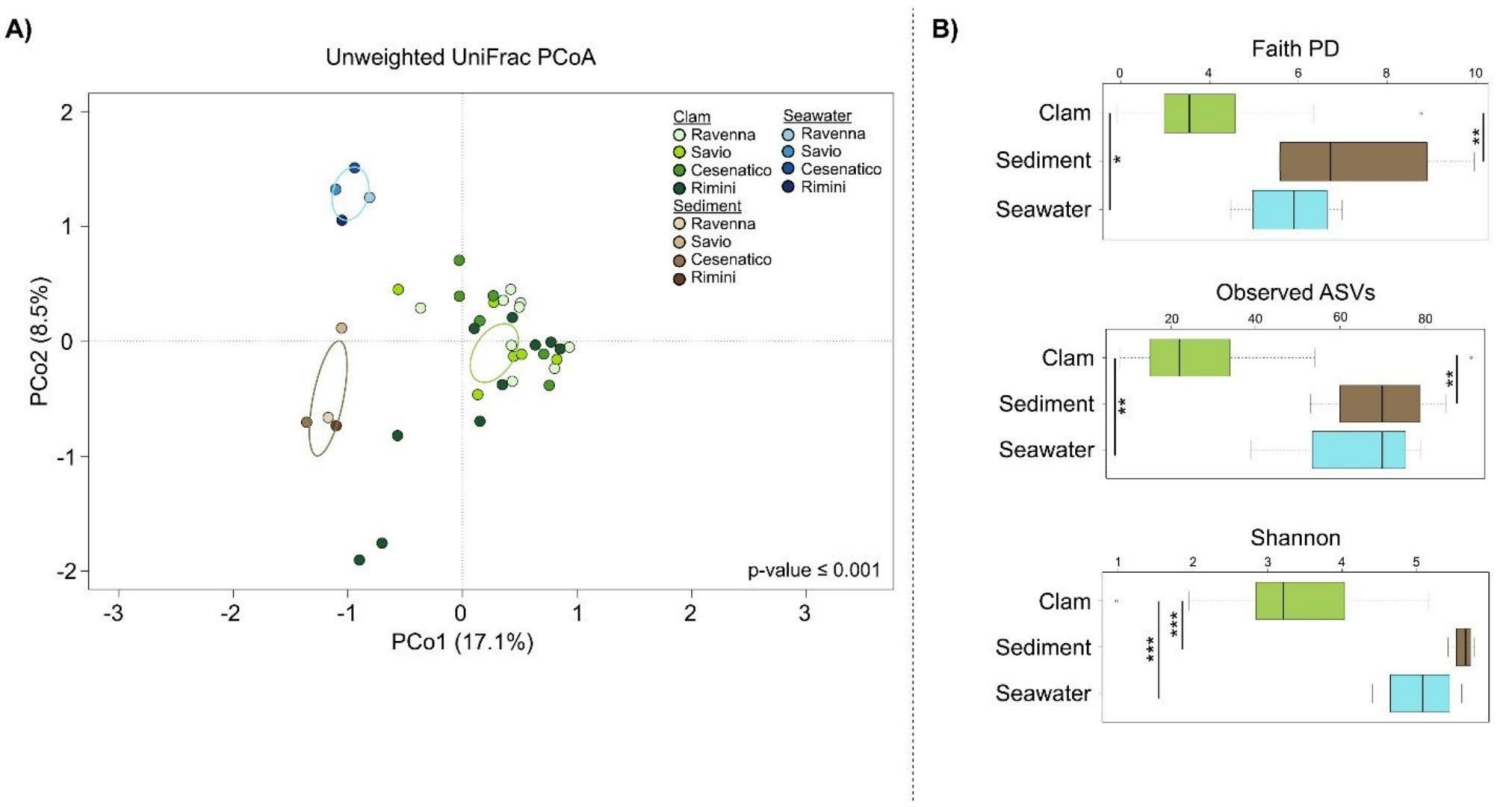
**Alpha and beta diversity of the*C. gallina*digestive gland-associated microbiome and the** surrounding **environmental microbiomes.** (**A**) Principal coordinate analysis (PCoA) based on unweighted UniFrac distances between the microbial profiles of *C. gallina* digestive glands and environmental samples (seawater and sediments) shows a significant separation between groups (permutation test with pseudo-F ratio, *p*-value ≤ 0.001). Color gradients indicate sampling sites (Marina di Ravenna, Lido di Savio, Cesenatico, and Rimini) according to the legend in the plot. The first and second principal components (PCo1 and PCo2) are plotted, and the percentage of variance in the data set explained by each axis is reported. Ellipses include a 95% confidence area based on the standard error of the weighted average of the sample coordinates. (**B**) Box-and-whiskers distribution of alpha diversity calculated using Faith’s phylogenetic diversity (PD), the number of observed ASVs, and the Shannon index. Significant *p*-values (Wilcoxon rank-sum test controlled for multiple testing using FDR) are indicated in the figure by the following symbols: * *p*-value ≤ 0.05; ** *p*-value ≤ 0.01; *** *p*-value ≤ 0.001

For each site, 36 out of the 50 collected clams were pooled on base 3, for a total of 12 pools per site, which were used to profile the DG-associated microbiome by 16 S rRNA amplicon sequencing. The microbial communities of corresponding sediments and seawater were also characterized. Sequencing was performed on a total of 48 clam pools, 4 seawater and 4 sediment samples, resulting in 6,677.4 ± 3,965.6 mean high-quality reads per sample (Suppl. Table S1) table and 1,056 ASVs. The microbial compositional structure at phylum and family level is shown in Suppl. Fig. S1. Overall, the clam DG-associated microbiome was dominated by the phyla *Bacillota* (42%) and *Planctomycetota* (18%), with *Pseudomonadota* (11%) and *Spirochaetota* (11%) as subdominant phyla. The seawater microbiome was dominated by *Pseudomonadota* (43%), *Actinobacteriota* (22%), *Bacillota* (17%), and *Bacteroidota* (14%), whereas the sediment microbiome was dominated by *Bacillota* (33%), *Pseudomonadota* (24%), and *Actinobacteriota* (18%). Alpha and beta diversity measurements of the described microbial datasets (Fig. 3) showed a clear segregation of clam-associated and environmental microbiomes (*p*-value ≤ 0.001 for beta diversity, permutation test with pseudo-F ratios), with the former showing lower levels of alpha diversity (*p*-value ≤ 0.05 for the alpha diversity comparison between clam and environmental microbiomes in all metrics, Wilcoxon rank-sum test; see Fig. 3 for further details). According to the PCoA plot, the clam DG-associated microbiome was closer to the sediment microbiome compared to seawater samples. These results confirm previous observations [36, 37] and emphasize the important connection between the clam-associated and sediment microbiomes. The separation in the PCoA also highlighted a certain heterogeneity of the clam DG-associated microbiome according to collection sites (*p*-value ≤ 0.05 permutation test with pseudo-F ratios, data not shown), confirming the observed site-specific features of the clam DG-associated microbiome [3, 38], even at a local scale. In Suppl. Fig. S2, we provide the compositional profile of the DG-associated microbiome at each sampling site. Despite a certain degree of site specificity, a core DG-associated microbiome of *C. gallina* was detectable, defined as the taxa with a prevalence higher than 70% in our sample set. This core included the phyla *Bacillota*, *Planctomycetota*, *Pseudomonadota*, and *Verrucomicrobiota*, and the families *Mycoplasmataceae*, *Peptostreptococcaceae* and *Pirellulaceae* as major components. On the other hand, no significant differences in the alpha diversity of the DG-associated microbiome were observed between sites (Suppl. Fig. S3). Finally, focusing on the sediment microbiome variation among sampling sites, we found that sediments from the high-CI site (Rimini) were mainly characterized by several microorganisms of environmental origin, both marine and terrestrial, such as members of the *Ilumatobacteraceae*, *Flavobacteriaceae*, *Hungateiclostridiaceae*, *Rhizobiaceae*, *Xanthobacteraceae* and *Rubritaleaceae*, with the exception of *Mycoplasmataceae*, that is known to be a host-associated microorganism [39]. Conversely, sediments from low-CI sites (Marina di Ravenna, Lido di Savio and Cesenatico) were enriched in host-associated or opportunistic microorganisms, such as *Lactobacillaceae*, *Streptococcaceae*, *Paenibacillaceae*, *Staphylococcaceae* and *Pseudomonadaceae* (Suppl. Table S2).

### Variations in the clam digestive gland-associated microbiome according to condition index

**Fig. 4:**
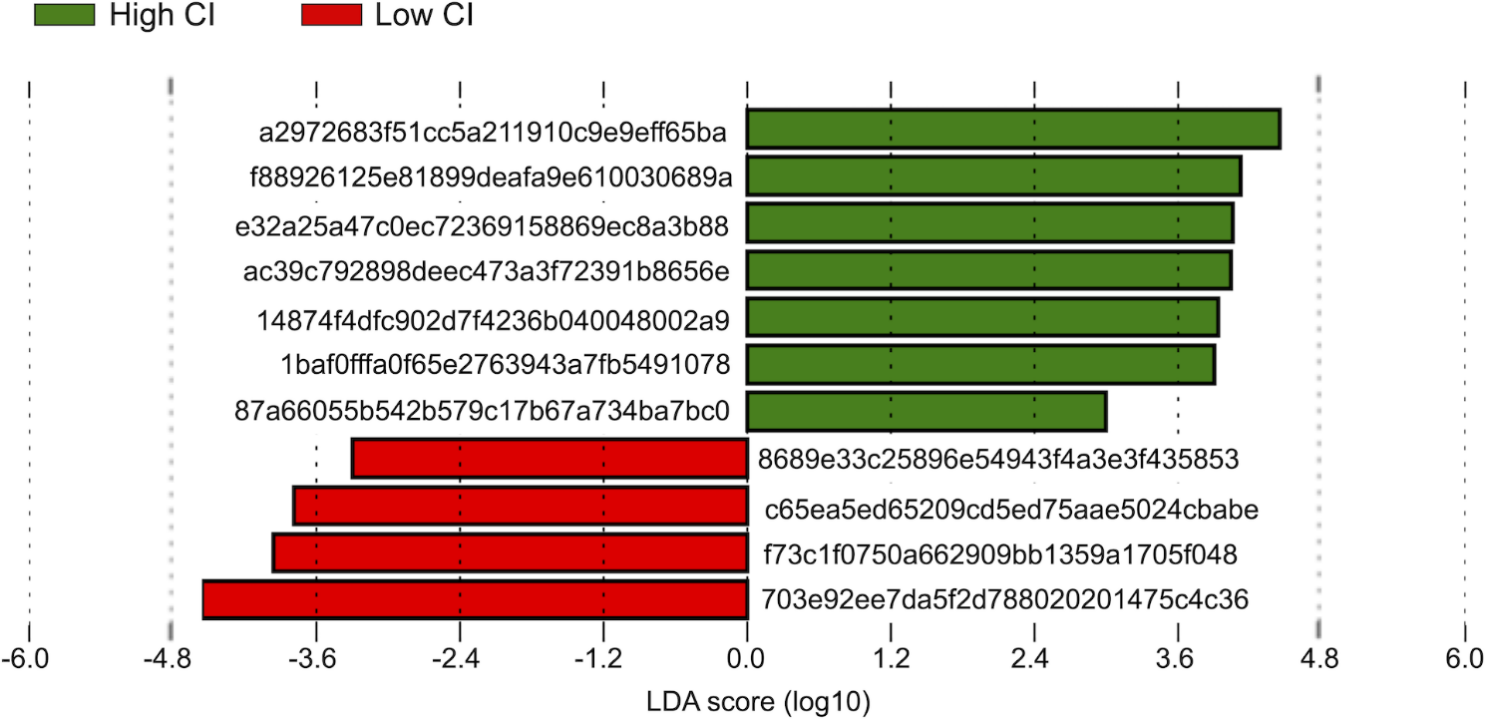
**Discriminant taxa between high- and low-CI sites.** Linear discriminant analysis (LDA) scores of discriminating ASVs between high- and low-CI sites. The plot was obtained by LDA effect size (LEfSe) analysis with the logarithmic threshold for discriminative features set to 2.0. Refer to Suppl. Table S3 for taxonomic assignment of the identified ASVs

To identify the compositional specificities of the DG-associated microbiome at high- and low-CI sites, we first applied LEfSe [31] at the ASV level (Fig. 4), and then used BLAST [32] to assign the corresponding bacterial species to the discriminant ASVs (Suppl. Table S3). According to our findings, the high-CI site was characterized by 7 discriminant species with a best hit corresponding to the species *Marvinbryantia formatexigens strain I-52* (percentage identity 93.10%, family *Lachnospiraceae*), *Prevotellamassilia timonensis strain Marseille-P2831* (94.88%, family *Prevotellaceae*), *Culturomica massiliensis strain Marseille-P2698* (93.27%, family *Odoribacteraceae*), *Duncaniella freteri strain TLL-A3* (86.67%, family *Muribaculaceae*), *Simkania negevensis strain Z* (90.32%, family *Simkaniaceae*), *Bacteroides oleiciplenus YIT 12,058* (97.12%, family *Bacteroidaceae*) and *Mariniblastus fucicola strain FC18* (97.77%, family *Pirellulaceae*). Low-CI sites were characterized by 4 discriminant taxa with a best hit corresponding to the species *Mycoplasmopsis mustelae strain MX9* (90.61%, family *Mycoplasmataceae*), *Mycoplasma procyoni strain LR5794* (88.73%, family *Mycoplasmataceae*), *Roseibacillus ponti strain YM27-120* (91.67%, family *Verrucomicrobiaceae*) and *Mariniblastus fucicola strain FC18* (94.74%. family *Pirellulaceae*). Discriminant ASVs ranged between 0 and 54% of relative abundance in the DG of clams from the different sites.

**Fig. 5:**
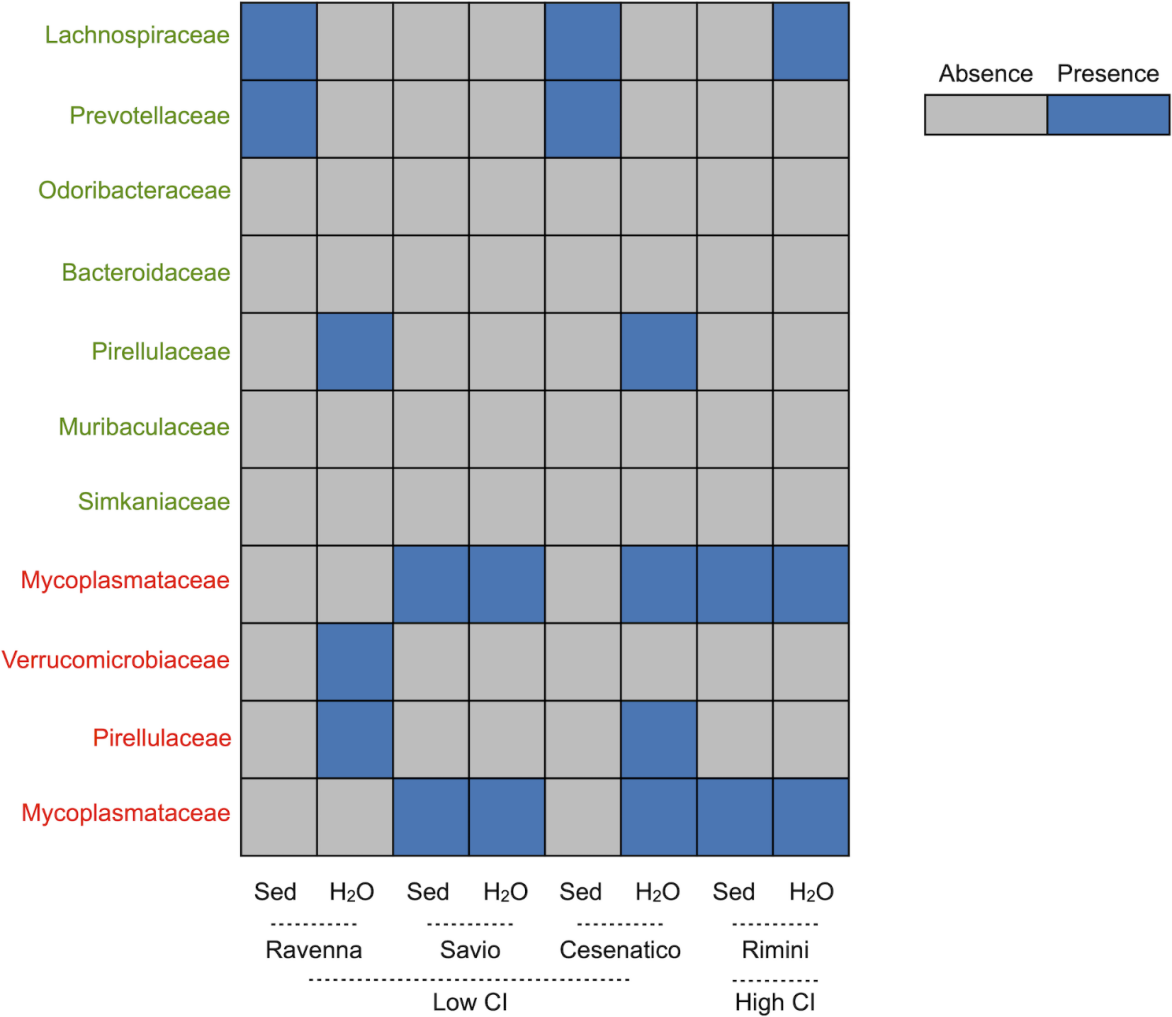
**Presence of digestive gland-associated microbiome taxa discriminating between high- and low-CI sites in corresponding environmental samples.** Presence (blue) or absence (grey) display of discriminant taxa identified by LEfSe in sediment (Sed) and seawater (H_2_O) microbiomes from high- (Rimini) and low-CI sites (Marina di Ravenna, Lido di Savio and Cesenatico). Discriminant taxa identified for the high-CI site are shown in green, those for the low-CI sites in red

To highlight the possible connections between these DG-associated microbiome components and the respective environmental ecosystem, we investigated the distribution of the respective families in the corresponding water and sediment microbial ecosystems from high- and low-CI sites (Fig. 5). Overall, the bacterial families corresponding to DG-associated species characterizing the high-CI site were sporadic in the environmental microbiomes, with only *Lachnospiraceae* present in the Rimini seawater, *Lachnospiraceae* and *Prevotellaceae* in the Ravenna and Cesenatico sediments, and *Mariniblastus* in the respective seawater. Conversely, bacterial families corresponding to DG-associated microbiome species characterizing low-CI sites were most pervasive in the environmental microbiomes. For instance, *Mycoplasmataceae* were quite pervasive in seawater and sediments from Savio, Cesenatico and Rimini. *Verrucomicrobiaceae* and *Pirellulaceae* were detected in Ravenna seawater, with *Pirellulaceae* also detected in Cesenatico seawater.

**Fig. 6:**
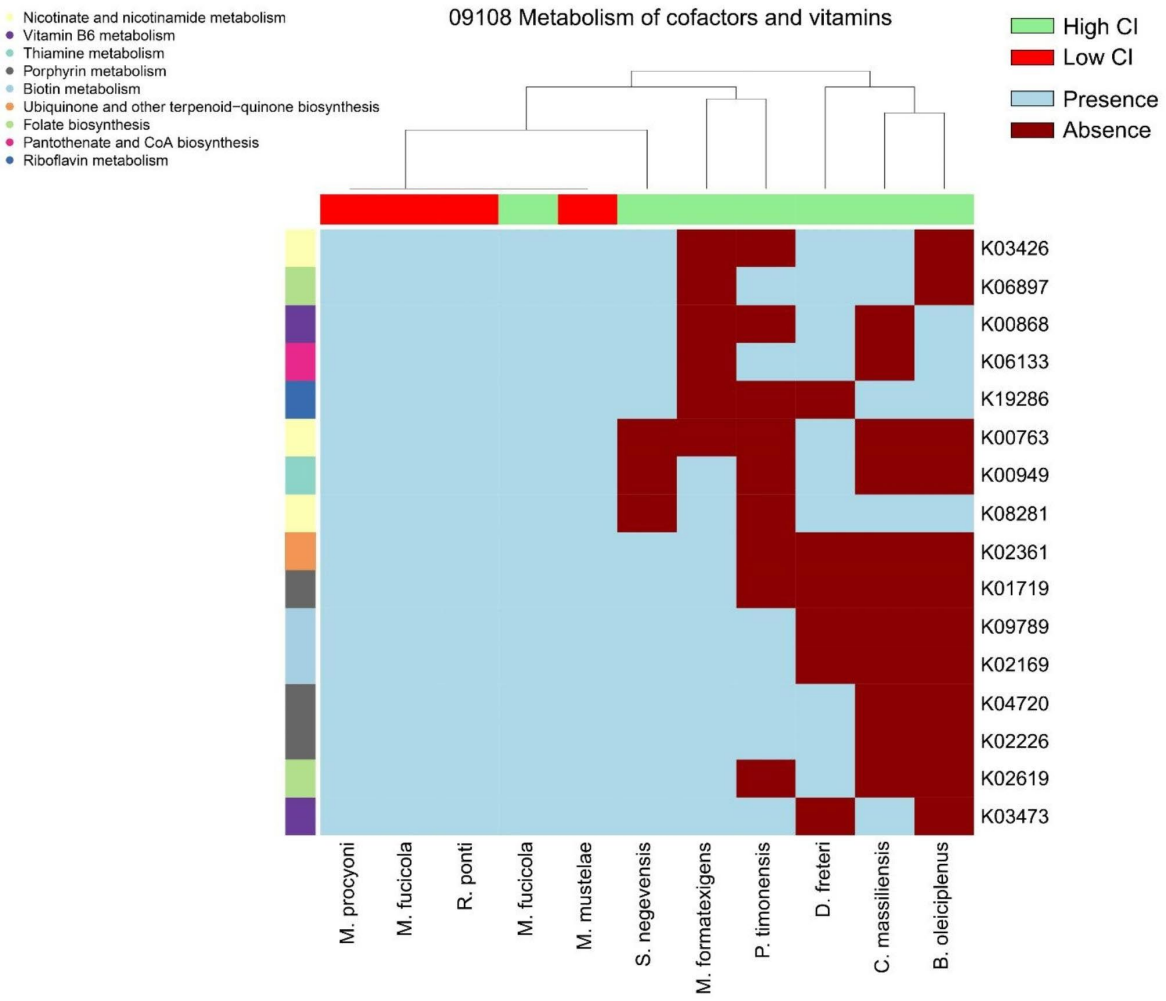
**Metabolism of cofactors and vitamins in high- and low-CI sites.** Heatmap representing the presence/absence of KO copy number in each discriminant ASV identified by LEfSe in the metabolic pathways of cofactors and vitamins

The functional features of the discriminant species identified for high- and low-CI sites were inferred using PICRUSt2 [33]. To emphasize the respective specificities while excluding core functionalities, only exclusive functions, defined as KO_ASVs, for at least 2 species for each discriminant group were considered. According to our findings, DG-associated microbiome species discriminating high- and low-CI sites showed different metabolic propensities, especially for pathways involved in carbohydrate, lipid, amino acid, nucleotide, and energy metabolism (Suppl. Fig. S4). Interestingly, only DG-associated microbial species characterizing the high-CI site were endowed with pathways involved in the metabolism of cofactors and vitamins, including, among others, the biosynthesis of pyridoxine (vitamin B6), folate, riboflavin and terpenoids, suggesting their possible role as health-promoting bacteria (HPB) (Fig. 6).

### Proof-of-concept experiment in controlled environment

In order to further explore possible connections between the clam DG-associated microbiome components, the surrounding sediment microbial communities and animal health, experiments were conducted under controlled conditions. We tested the hypothesis that sediments from the high-CI site (i.e., Rimini) would favor the increase of health-promoting microorganisms in clams from a low-CI site (i.e., Ravenna), possibly resulting in an improved physiological status of the animal. To this end, clams collected in Ravenna were reared in aquaria on both Ravenna and Rimini sediments. The DG-associated and sediment microbiomes were assessed at 3 time points: T0 (right after the experimental set-up and acclimatization), T1 (after 7 days of incubation) and T2 (after 21 days of incubation). For each aquarium, 15 clams were collected and pooled on base 3 at T0 and T1, and 9 clams were collected and pooled at T2, for a total of 52 clam pools, and 3 sediment sample were collected for each aquarium at all timepoints, resulting in 6,566.0 ± 3,368.4 mean high-quality reads per pool and 3,992 ASVs (Suppl. Table S4). Clams collected at T0 at T2 were used to assess CI values. According to our observations, Ravenna clams reared on Rimini sediments showed a better performance in terms of ΔCI when compared to their initial condition (Ravenna clams on Ravenna sediments). More specifically, the ΔCI between T2 and T0 for Ravenna clams reared on Rimini sediments was > 1 (ΔCI_Ra−Ri_ = 1.37), whereas for Ravenna clams reared on Ravenna sediments it was close to 0 (ΔCI_Ra−Ra_ = -0.15). Interestingly, when we assessed the total relative abundance of previously identified HPB (namely, *Marvinbryantia formatexigens*, *Prevotellamassilia timonensis*, *Culturomica massiliensis, Duncaniella freteri, Simkania negevensis*, *Bacteroides oleiciplenus* and *Mariniblastus fucicola*) in the clam DG-associated microbiomes in controlled environment, we observed that clams reared on Rimini sediments maintained a relevant proportion of these microorganisms up to T2 (570 RPKM, where Reads Per Kilobase per Million reads mapped were calculated by dividing the number of reads mapped to each reference sequence by the mean kilobase length of that sequence and the total number of reads in that sample times 1 million). Conversely, the identified HPB progressively disappeared in the clams reared on Ravenna sediments during the observation time (0 RPKM at T2). No relevant difference in the reads count of these microorganisms was observed between Ravenna and Rimini sediments. Taken together, our data suggest the possibility that sediments from Rimini may favor the physiological status of the clam, by promoting the acquisition of certain microorganisms, resulting in an overall improvement in animal health.

## Discussion

In this work, we explored the connections between the *C. gallina* DG-associated microbiome, the surrounding environmental microbiomes, and the nutritional status of the animal in 4 productive sites along the Emilia Romagna coast, stretching about 60 km along the Northwestern Adriatic Sea.

According to our findings, *C. gallina* individuals are able to select for a specific DG-associated microbiome, which is closely linked to the surrounding sediment ecosystem, while retaining a well-recognizable and host-selected compositional layout. Interestingly, we observed a relevant, site-specific degree of variability in the DG-associated microbiome, confirming previously reported variations in the DG-associated microbiome according to geography [3, 38, 40], but at a spatially smaller scale, as the mean distance between two consecutive sites was only about 20 km. Even in the context of such site-specific variability, we were able to identify a core DG-associated microbiome in *C. gallina* individuals up to the family level, including *Mycoplasmataceae*, *Peptostreptococcaceae* and *Pirellulaceae* as the most prevalent microbial families.

In general agreement with the available historical data [2] and according to the measured CI of the collected samples, the clams collected in Rimini showed an overall better nutritional state compared to those collected in Marina di Ravenna, Lido di Savio and Cesenatico. Interestingly, some DG-associated microbiome species were found to be specifically associated with either high- or low-CI sites, namely *Marvinbryantia formatexigens*, *Prevotellamassilia timonensis*, *Culturomica massiliensis, Duncaniella freteri, Simkania negevensis*, *Bacteroides oleiciplenus* and *Mariniblastus fucicola strain* with the high-CI site (i.e., Rimini) and *Mycoplasmopsis mustelae*, *Mycoplasma procyoni*, *Roseibacillus ponti* and *Mariniblastus fucicola* with the low-CI sites. Based on the functional features predicted for these discriminating taxa, we observed that the DG-associated microbial components characterizing the high-CI site were equipped with functionalities for the biosynthesis of cofactors and vitamins, such as pyridoxine, folate, riboflavin and terpenoids, suggesting their possible role as HPB. Indeed, they could provide the host with essential nutrients, and enhance its defense through the production of molecules such as terpenoids [41–43]. In contrast, *Mycoplasmopsis* and *Mycoplasma* species, characterizing the DG-associated microbiome of low-CI sites, have been, respectively, associated with distemper-like symptoms in mink [44] and showing high sequence similarity with a nearly complete 16 S rRNA gene of a *Mycoplasma* strain cultured from a racoon clinical sample [45]. These findings are in line with the evidence of *Mycoplasma* and *Mycoplasmopsis* species for a strong propensity in causing chronic infections in humans and other vertebrates, effectively bypassing host immune responses [46, 47]. For example, members of the family *Mycoplasmataceae* have been associated with disease development in susceptible oysters [3].

When investigating the presence of DG-associated microbial taxa discriminating high- and low-CI sites in the respective environmental ecosystems, we observed that taxa discriminant for low-CI sites were generally pervasive in sediment and seawater microbiomes, whereas taxa discriminant for the high-CI site showed a more sporadic behavior, being rarely detected in environmental microbiomes. Finally, through experiments under controlled conditions, we were able to provide some preliminary evidence on the role of sediments from the high-CI (Rimini) site in favoring the possible acquisition of a probiotic DG-associated microbiome configuration by clams. Indeed, Ravenna clams reared on Rimini sediments showed an increase of the previously identified HPB and a better performance in terms of CI variation over time, compared to the same clams reared on Ravenna sediments.

## Conclusions

Taken together, our data suggest the possible existence of an indirect support mechanism from sediment microbiomes in the acquisition of HPB by the clam DG-associated microbiome, resulting in a better physiological and nutritional status of the host. Further investigation, in particular the implementation of tailored metabolomic approaches (e.g., LC/GC ultra-high resolution mass spectrometry), carried out in parallel with CARD-FISH experiments, allowing the visualization of HPB in the native ecosystem, could help shed light on these mechanisms, supporting our evidences indicating that specific microbial components in marine sediments (i.e., the high-CI site Rimini) may provide molecular modulators that interact with the microbiome of *C. gallina*, thus promoting a higher HPB abundance in the DG-associated microbiome. Such molecular modulators may include micro and macro nutrients or substrates facilitating the growth of probiotic microbiome components [3, 48], with potential beneficial effects on the host animal by improving its microbial balance [49]. Conversely, sediments from the low-CI sites, e.g., Ravenna, may negatively interact with DG-associated microbial components due to the presence of specific xenobiotics compounds, such as hydrocarbons and heavy metals [50–52], as already observed in Venice lagoon area [3] and the Abruzzo coast [3].

Health promoting bacteria might contribute to the host health by providing essential nutrients, such as vitamins and cofactors, but also defense molecules, essential for controlling opportunistic pathogens and keeping an eubiotic configuration of the DG-associated microbiome. Our findings may contribute to the definition of innovative microbiome-based management strategies for the preservation of *C. gallina* productivity in the Adriatic Sea, in particular for the retention of a probiotic niche at the sediment-animal microbiome interface.

### Electronic supplementary material

Below is the link to the electronic supplementary material.

Supplementary Material 1

Supplementary Material 2

## Data Availability

Processed reads for 16 S rRNA amplicon sequencing are openly available in European Nucleotide Archive (ENA), reference number PRJEB64620.
