# Supplementary material for: Microbiome variation at the clam-sediment interface may explain changes in local productivity of Chamelea gallina in the North Adriatic sea
Source: BMC Microbiol. 2023 Dec 19;23:402. doi: 10.1186/s12866-023-03146-8 (PMC10729368; doi:10.1186/s12866-023-03146-8)
Supplement: Supplementary file 2 — Supplementary Material 2 [file 12866_2023_3146_MOESM2_ESM.pdf]

Microbiome variation at the clam-sediment interface may explain changes in the local productivity of *Chamelea gallina* in the North Adriatic Sea

Giulia Trapella[1,4], Nicolò Cinti[1], Luca Parma[2], Antonina De Marco[2,4], Andrea Nicolò Dell'acqua[1,4], Silvia Turroni[1], Simone Rampelli[1,4], Daniel Scicchitano[1,4], Letizia Iuffrida[3,4], Alessio Bonaldo[2], Silvia Franzellitti[3,4], Marco Candela[1,4] and Giorgia Palladino[1,4]

Supplementary figures

[1]Unit of Microbiome Science and Biotechnology, Department of Pharmacy and Biotechnology (FaBiT), Alma Mater Studiorum – University of Bologna, 40126 Bologna, Italy

[2]Department of Veterinary Medical Sciences, University of Bologna, 40064 Ozzano Emilia (Bologna), Italy

[3]Animal and Environmental Physiology Laboratory, Department of Biological, Geological and Environmental Sciences (BiGeA), University of Bologna, 48123 Ravenna, Italy

[4]Fano Marine Center, The Inter-Institute Center for Research on Marine Biodiversity, Resources and Biotechnologies, 61032 Fano, Italy

A)

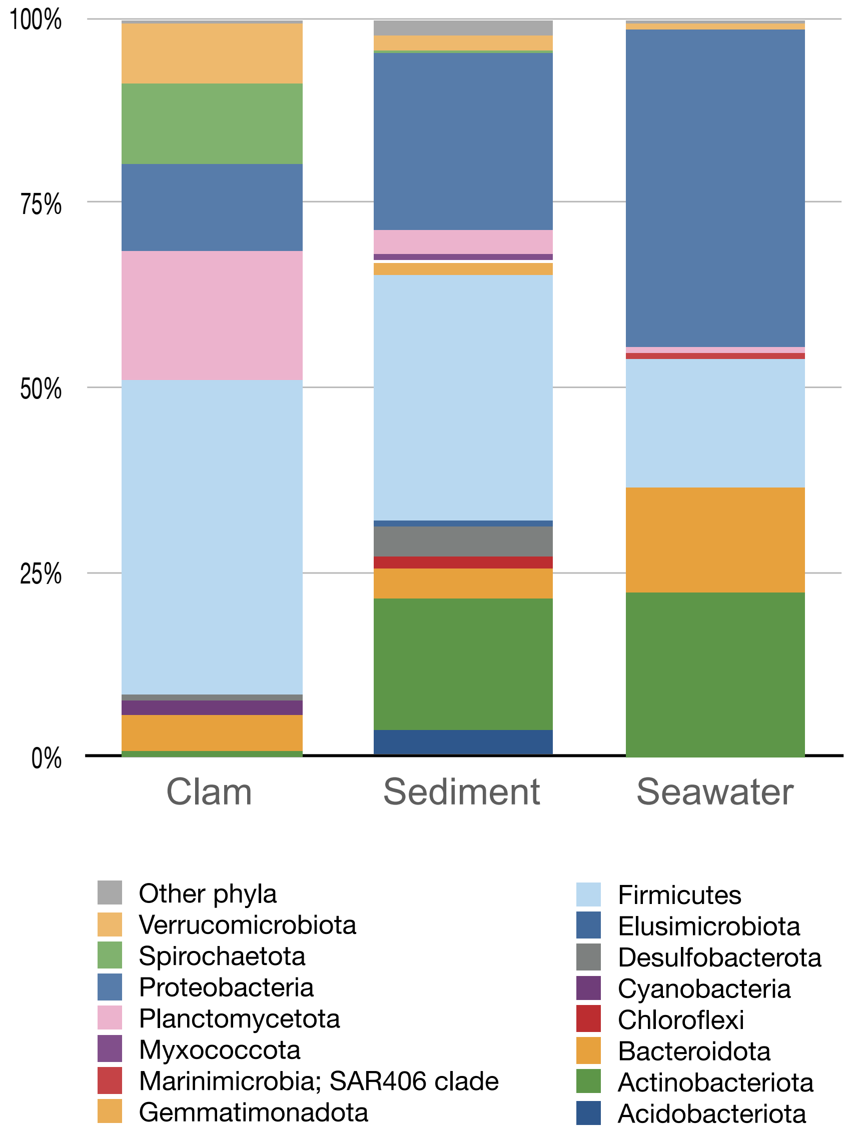

B)

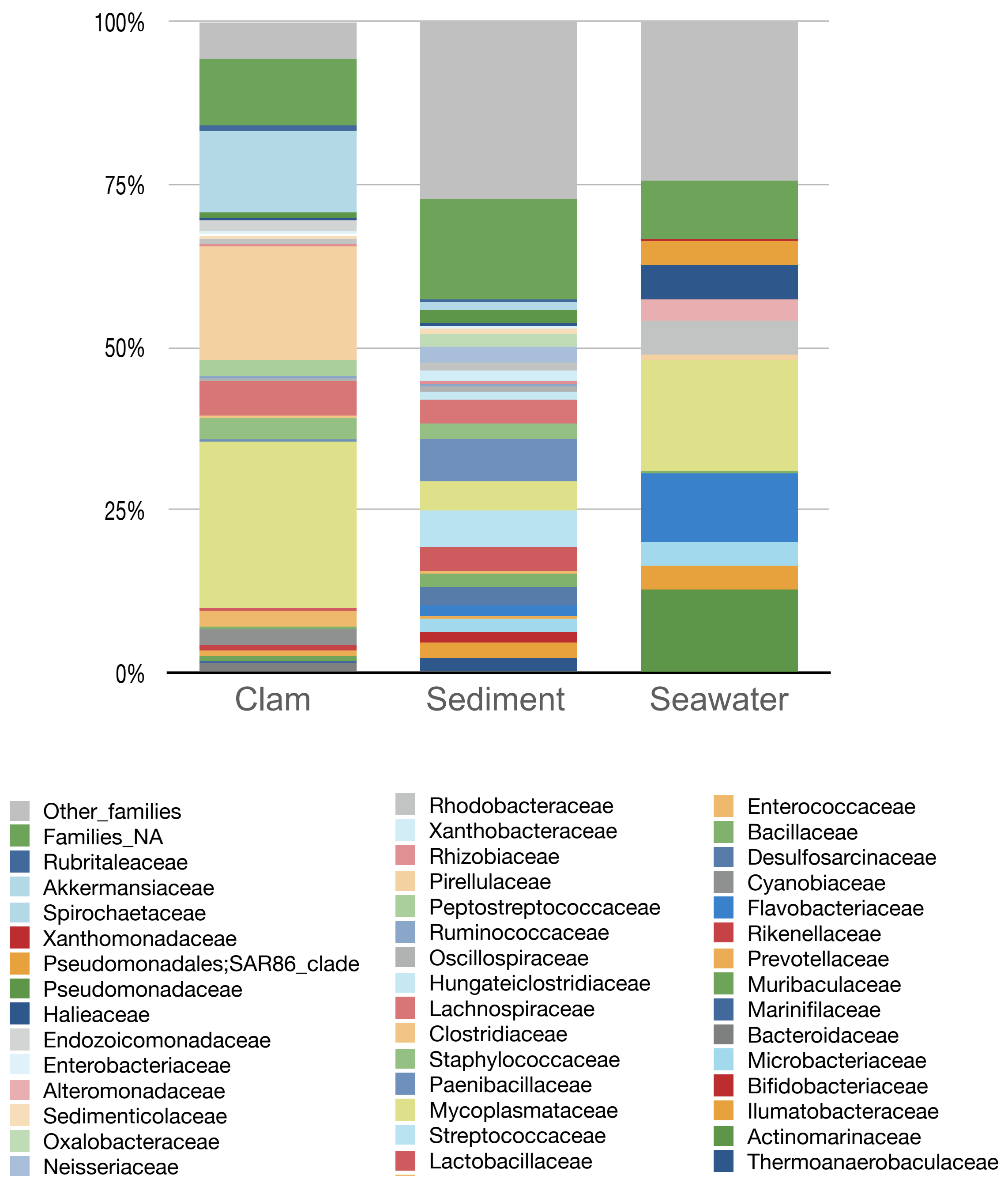

Supplementary Figure S1 - Microbial communities associated with clam digestive glands, sediment and seawater in the North Adriatic Sea at phylum and family level. Barplots summarizing the overall phylum (A) and family (B) level microbial composition of the different ecosystems, namely clam, sediment, and seawater. Phyla with relative abundance > 2% and families with relative abundance > 5% in at least 1 sample are represented. NA = not assigned.

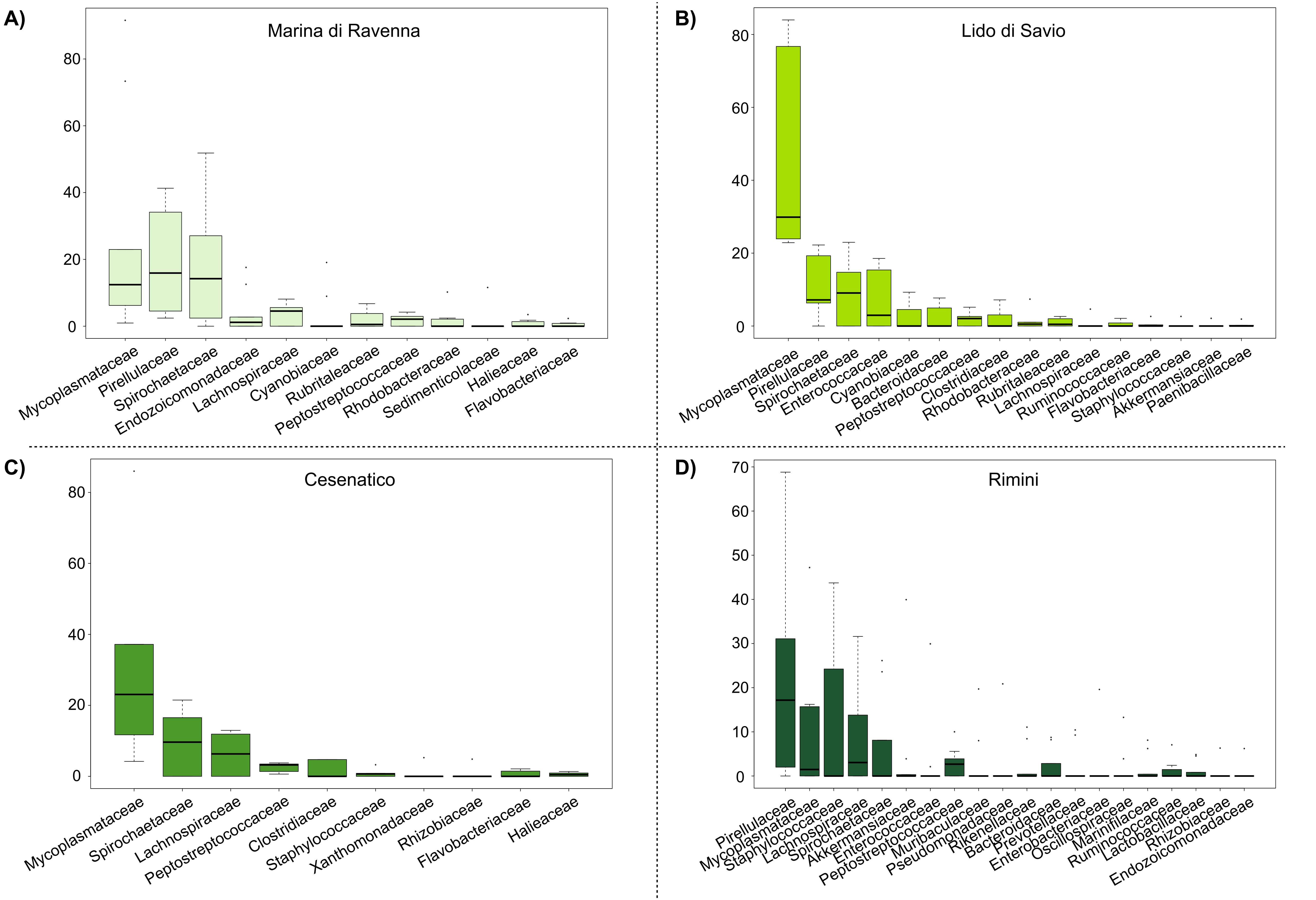

Supplementary Figure S2 - Microbial communities associated with clam digestive glands from 4 production sites in the North Adriatic Sea at family level. Boxplot summarizing the DG-associated microbial composition at the family level at the different sampling sites. Families with mean relative abundance (r.a.) > 0.5%, 0.4%, 0.5% and 0.6% for Marina di Ravenna, Lido di Savio, Cesenatico and Rimini, respectively, are represented. Different r.a. values were selected for representation purposes.

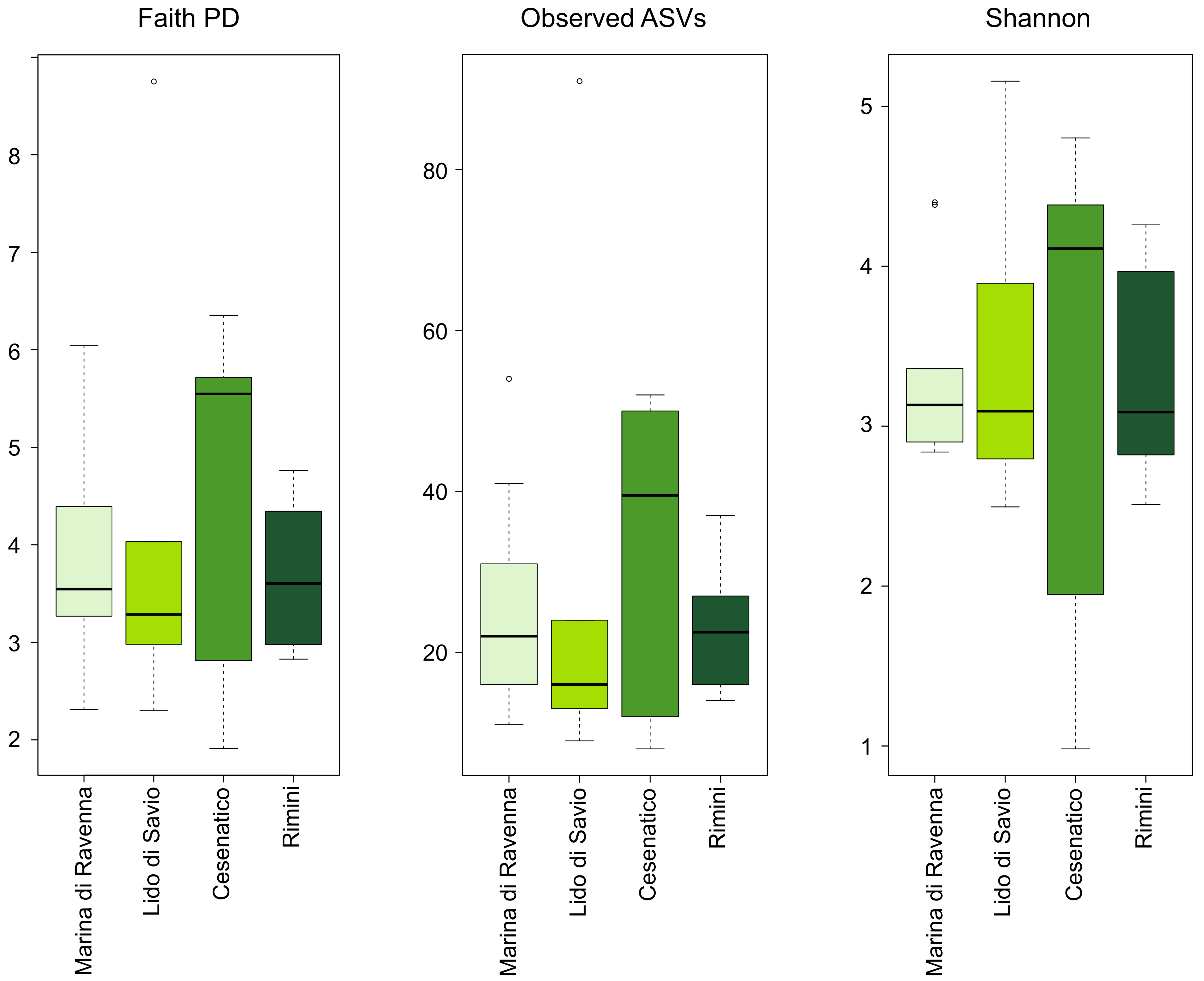

Supplementary Figure S3 - Box-and-whiskers distribution of alpha diversity of *C. gallina* digestive gland-associated microbiome in the different sampling sites. Faith's phylogenetic diversity (PD), the number of observed ASVs, and the Shannon index are represented. No significant p-values (Wilcoxon rank-sum test controlled for multiple testing using FDR) were found.

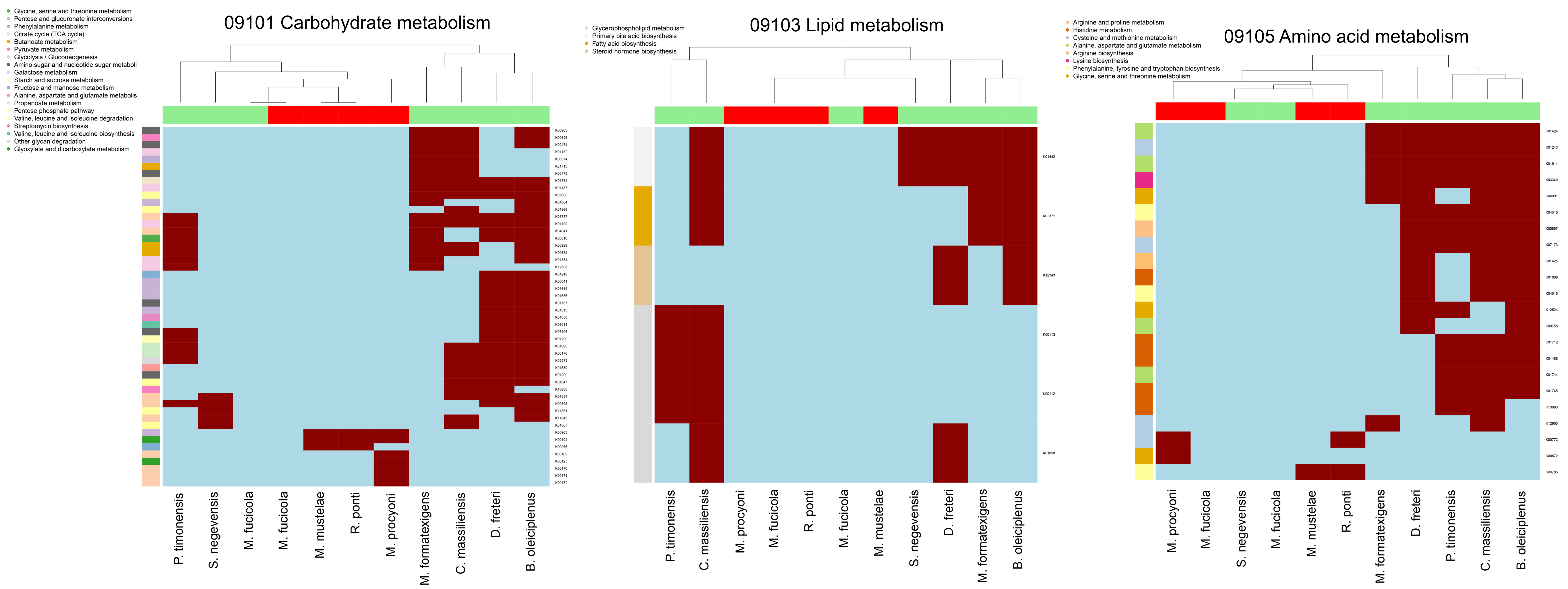

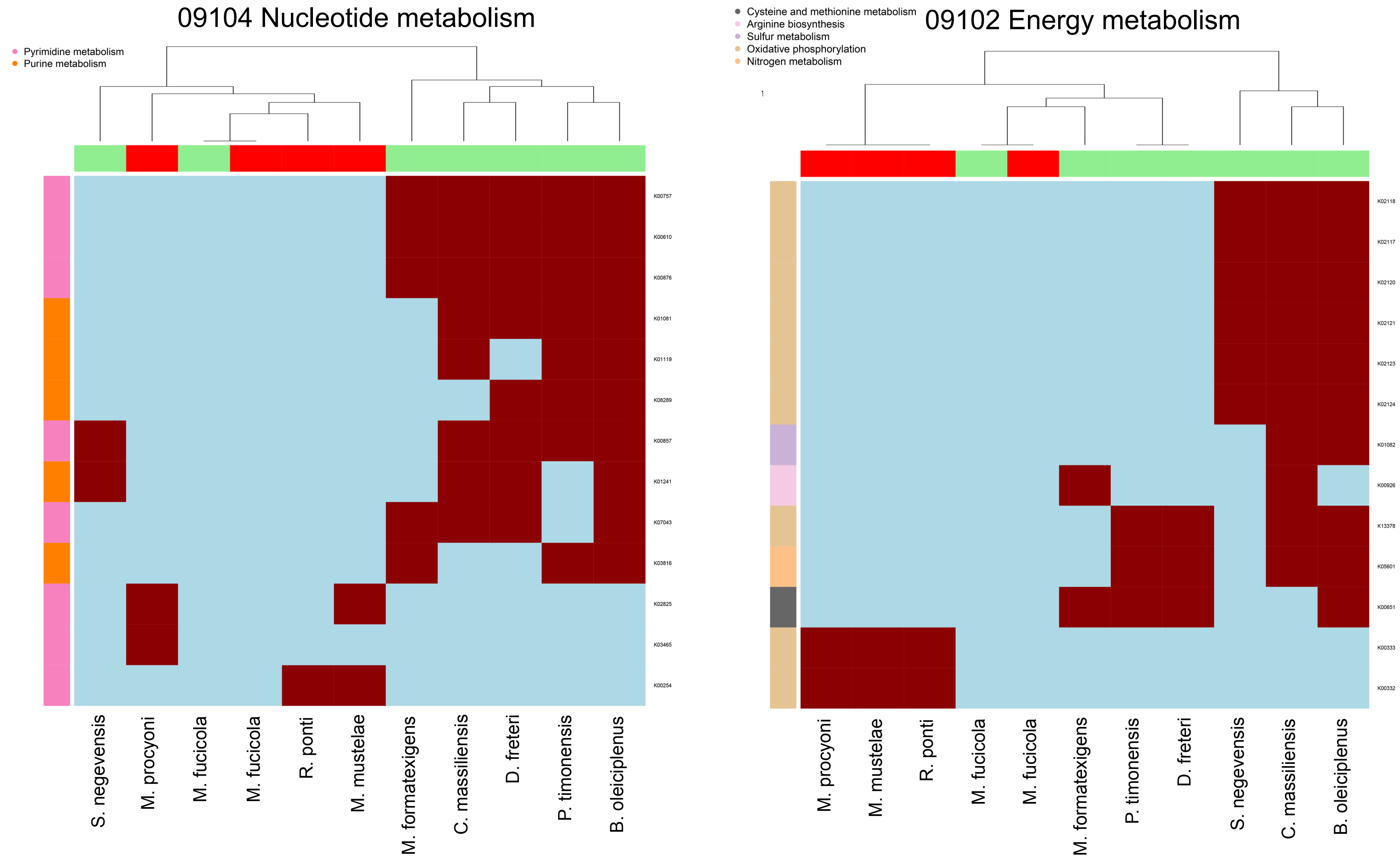

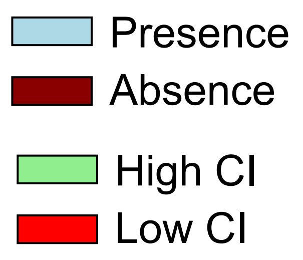

Supplementary Figure S4 - Metabolic propensities of high- and low-CI sites. Heatmaps representing the presence/absence of KO copy number in each discriminant ASV identified by LEfSe. The reference pathway is shown on top of each heatmap. Reference pathways for each KO are reported in the color code legend on the left of each heatmap. CI = condition index.
